# Nomenclature

**Published:** 1877-11

**Authors:** 


### NOMENCLATURE.

At the meeting of the American Dental Association, held at Chicago in August last, a committee was appointed on Nomenclature and Terminology.

That there is a demand for work in this direction there is no doubt.

The medical lexicons fail in many particulars to answer the requirements of dental science.

The only dental dictionary we have is that of the late Dr. C. A. Harris. This is a good work, and should be in the hands of every dentist.

It is invaluable to the dental students. The names and terms embraced in his preparatory course will be found there. This work, full as it is, does not, up to this time, embrace everything. New words and terms are being introduced, and in some instances old ones are perverted and improperly used. The object of this committee is to examine and place in their true position, so far as definition and use are concerned, those words and terms that are misused, at least so far as they may be brought to the attention of the committee. It is desirable that all who have questions or suggestions in mind on this subject, present them to some member of the committee. It consists of Drs. H. Judd, of St. Louis, Mo., W. H. Atkinson, of New York, and J. Taft, of Cincinnati, O.

